# Legal scenario in burn care in India

**DOI:** 10.4103/0970-0358.70729

**Published:** 2010-09

**Authors:** Atul Kumar Shah

**Affiliations:** Convener, Legal Cell, Association of Plastic Surgeons of India and Consultant Plastic Surgeon, Vadodara, India

**Keywords:** Burn care, medico legal

## Abstract

Physicians engaged in management of burn patients in India need to keep themselves abreast with the legal requirements. Clinical burn management and liaison with local authorities go almost parallel. Concept of the legal rights of Burn Survivor and the family are emerging now in India. Demarcation between physical impairment status and disability to sustain are discussed. Burn Physicians can help their patients by imparting this information. Pertinent details about Workmen’s compensation act, Persons with disabilities act and guidelines for calculation of physical impairments are listed.

## INTRODUCTION

Usually, burn accidents take place due to failure to prevent them. This trauma has an element of suspicion of a crime added to it in many incidences. A treating physician has the responsibility of informing such accidents to legal authority. Legal understanding is not consistent and proceedings are sometimes lengthy.

## SOCIAL, CIVIC AND MORAL DULY

Except as required in section 39 (section 40 for village head, police associate or a villager) of the Criminal Procedure Code, the need of informing the police in case of a suspicious burn injury is a social as well as a moral responsibility.

The following situations need to be notified to the police.

1. All major burns when received
2. Unexplained severity, not matching with the history or circumstances
3. Patients received after several days of burns
4. Patients received without proper treatment
5. Patients likely to succumb to the injury
6. Patients received dead
7. Mass casualties

## INDIAN LEGAL SCENE

Indian Penal Code enlists various body injuries. Code of Criminal Procedures in section 39 asks every citizen to inform the police of any incidence which has harmed any human being. For the treating physician, informing the police is thus an extension of his duty as a citizen. To the best of the knowledge of author, there is no requirement or compulsion specified in Medical Council of India (MCI) Act or Bombay Police act and the like.

Usually, in a majority of treating places in India, when a patient succumbs to the injuries, certificate of death is required to be issued. In this certificate, a cause of death has to be specified. When the treating doctor is not certain about the cause of death, demand of a medicolegal post mortem has to be made. Such a demand can only be made through a police officer. When a police officer is informed of such a request, his team has to visit the site where death has occurred. Police Officer in charge of the case, writes down a report of the condition and situation of the body, known as “panchnama”. He also fills an inquest for a medico-legal post mortem. Later, the body is handed over to this police officer, who takes it to nearby health establishment equipped with post mortem facilities. If the inquest was filled in odd hours, the body is shifted to a morgue for later post mortem, or the body may be retained at the request of the police officer, in the same room, till it is shifted for post mortem the next day morning. The police will enter the scene and fill inquest without wasting time, only if they had been informed when the patient first came for treatment or got admitted.

Sometimes, patients come from distant places, and as per the police codes, the police officers of the area of incident are required to carry out legal procedures. It is important to inform the nearby police immediately so that at the time of the death of the patient, the nearby police do not waste time in procedural delays.

### Burn injuries in a married female

When a married woman is brought with extensive burn injuries, the police tend to treat this mishap as a dowry problem unless proved otherwise with circumstantial evidences. If the married life at the time of mishap is less than 10 years, then the police investigation becomes stringent and is under a senior officer of rank not less than a deputy commissioner. Death in such cases would be governed by section 304B of Indian Penal Code which specifies dowry deaths and can lead to imprisonment ranging from 7 years to life.

### Disability and disfigurement

Burn disfigurement brings unwanted stress and breaks down self-confidence and self-esteem. Burn injury victims also suffer excruciating pain as their wounds heal. Burn patients undergo painful skin grafting surgeries to repair the damage done by the injuries. Burn survivors may endure physical trauma, pain, body metabolism damage, and changed temperature sensitivity. After the physical scars begin to heal, the emotional scars begin to surface.

Burn accidents could be caused by someone’s carelessness or failure to act. Often, it is a combination of negligent act or a failure to act by one or more persons or entities that cause an accident to occur. Burn injuries can result from a variety of different activities and sources. Whether at home or on the job one can be exposed to unknown dangers. The law requires that each person owes every other person a duty to act as a reasonably prudent person would act under the same or similar circumstances. When a person or entity does not act reasonably, then they have violated that duty and will be held responsible for any injury or damage that results. This duty is created from statutory laws or prior judgments of the cases that have evolved over time.

As burn specialists, we must have some understanding of the legalities of the award of disability by various competent authorities. In India, currently we have to depend mainly on the Workmen’s Compensation Act of 1923 and the Motor Vehicle Insurance Act to understand the concept. Physical impairment may translate into limitations of functional capacity of any individual and these limitations may be termed as “disability to sustain” or “disability to earn livelihood”. The definitions from World Health Organization (WHO) may make this point clear.

### Impairment and disability

#### Impairment

This may be permanent or transitory, psychological or anatomical loss and/or abnormality. Paralysis amputation, diabetic retinopathy and even burn disfigurement or contracture are the physical impairments.

#### Functional limitations

The functional limitations are partial or total inability to perform the activities necessary for motor, sensory or mental functions within the range and manner of which the human being is normally capable, such as walking, lifting loads, seeing, speaking, reading, writing, counting, taking interest in and making contact with surroundings. Functional limitations come from physical impairment and may last for a short time, a long time, be permanent or reversible. The idea is that whenever possible, one must be able to quantify these functional limitations. So, these limitations are described as regressive or progressive.

#### Disability

Disability is the difficulty that exists in performing one or more activities. These are the “activities” in accordance with the age, gender and normative social role of the person, and are generally accepted as essential and basic components of daily living such as self-care, social relations and economic activity. Physical Impairment and functional limitations are the causative factors for this disability. Also depending in part on the duration of functional limitation, disability may be permanent, long-term or short term. We may, however, be dealing with Permanent Physical Impairment (PPI) and Permanent Disability most of the time.

Recently, WHO has proposed revision of the earlier definitions and gave new terminologies like “activity limitation” and “participation restriction”. Activity is the nature and extent of functioning at the level of the person and may be limited in nature, duration and quality. Participation is the nature and extent of a person’s involvement in life situation and the restriction is in relation to impairment of activities, health conditions and contextual factors, and participation may be limited in nature, duration and quality.

Disability is defined in Webster’s Dictionary as

- state of being disabled: absence of competent physical, intellectual, or moral power, fitness or the like; also an instance of such lack
- legal incapacity, incompetence, or disqualification.

There are physical, social, psychological and vocational effects to disability as well. A trauma specialist is thus not equipped and qualified to determine the disability of a person, since disability is not purely a medical condition. Medical doctors must therefore restrict to evaluating and certifying physical impairment. In doing so, the medical expert wishfully thinks that the competent authority shall subsequently consider the social, psychological and vocational aspects in calculating the disability of the individual in question. Unfortunately, the compensation authorities or the courts of law currently have different understanding in demarcating between physical impairment and disability, mainly brought from the presentations and discussions of defending insurance counsels. Facial burns may not be considered a disability at all, but how can such a person ever become a marketing executive or a model or a TV news reader! On the other hand, a paraplegic is considered highly disabled, but he earns fortune by his vocal performances or lecturing on stress management and also qualifies for the concessions available to a disabled!

Majority of burn injuries are either domestic or vehicular. If it is part of work accident, then Workmen’s Compensation Act can be attracted. Under the Workmen’s Compensation Act, disability may be divided into three periods.

#### Permanent disability

Shall apply to permanent damage or to loss of use of some part of the body after the stage of maximum improvement has been reached and the remaining physical impairment is stationary, this physical impairment shall then be termed as permanent physical impairment.[1] There is a question of time frame here, and one must allow at least 6 months, if not more, before quantifying permanent physical impairment.

#### Temporary total disability

Shall be the period during which the individual is under treatment and totally physically impaired for the time being.

#### Temporary partial disability

Shall be the period when the physical impairment is partial to begin with or the person has recovered from the total physical impairment.

Ultimate legal compensation may be awarded usually for the permanent physical impairment of nonreversible nature, leading to permanent disability. A medical person thus needs to evaluate permanent physical impairment and may even be called by the court of law to testify as an expert witness. Physical impairment certificates can be issued by all medical graduates who are registered under schedule I of MCI act 1956, and there is no need for the medical graduate to be a specialist, as is sometimes demanded by the lawyers. However, the court always considers the specialisation and the expertise of the certifying doctor while relying on the assessment of physical impairment certified by the doctor.

In India, apart from the Workmen’s Compensation Act,[2,3] the only other authentic documents available till date to the knowledge of the author are The Manual for Doctors to evaluate Permanent Physical Impairment,[4] and Evaluation of Impairments, Disabilities in case of Occupational and other Diseases and Accidents.[5] The contents of the Manual for Doctors to evaluate Permanent Physical Impairment are based on the experts’ group meeting on disability evaluation that was held in New Delhi in September 1981, and also the National Seminar on Disability Evaluation and Dissemination held in New Delhi in December 1981. The manual is endorsed by DGHS, AIIMS and WHO and was published in March 1982. The evaluation criteria are simplified and can be of immense help in the issue of certificates of Permanent Physical Impairment.

## PLIGHT OF A BURN SURVIVOR

When any earning member of the family sustains major burn injury, the entire family receives the crunch. Unlike other trauma or disease, the management of burns is long, painful and costly. The economic resources of the family get exhausted with the extent of treatment. Industrial burn accident victims are no exception. The burn survivor has to face challenges constantly even after the initial assault is over. These are listed as follows:

1. Physical and emotional trauma,
2. An injury that leaves one in pain,
3. Disfigurement,
4. Organ damage,
5. Metabolic and biochemical damage and
6. Sensitivity to temperature change.

## LEGAL RIGHTS OF BURN SURVIVORS TO COMPENSATION

Highly dependant upon the prevailing legal and social support, the following compensation can be claimed by the burn victim depending on the cause of the burn injury. Compensatory damages “compensate” the injured person for various kinds of losses or damages, and include current medical expenses, lost wages, anticipated future medical expenses, anticipated future loss of wages, mental or emotional pain and suffering (past and anticipated in the future), disfigurement, and any physical or mental impairment or disability.

- Payment for treatment past and future
  Hospitalisation
  Surgical procedures
  Ongoing medical cares
  Counselling
  Scar revisions – cosmetic surgery
  Physical therapy
  Occupational therapy
- Compensation for loss of income – past and future
- Vocational rehabilitation (job retraining)
- Compensation for pain and suffering – burn survivors may be entitled to compensation for the pain and suffering that they have endured and may continue to endure as a result of their injury
- Loss of consortium – This is a novel concept for Indian Judiciary and may soon find its place in the case laws. The spouses of a burn survivor may be entitled to compensation when an injury is so severe that it interferes with the injured party’s spousal relations. The affected family member may suffer a very real detriment. Many courts recognise the right of the injured party’s spouse to recover in an appropriate case for a loss of support, services, love, companionship, society, affections, sexual relations and solace in the form of a loss of consortium action. Loss of consortium is a claim separate from the injury victim’s claim. It is unique to the injured party’s spouse and is compensable by a separate damages recovery.

### Indian legal enforcements

Burn survivors with untreatable contractures, keloids, grafted area, disfigurement and the like can be qualified to receive state help. Burn and trauma experts need to put up united front in making these benefits available to burn survivors. The enactment of the “Persons with Disabilities (Equal Opportunity, Protection of Rights and Full Participation) Act, 1995 (referred to as Persons with Disabilities Act), is a signal achievement of the Indian disability movement. Preamble to this act clearly delineates its objectives of promoting and ensuring equality and full participation of persons with disability. The act aims to protect and promote economic and social rights of people with disability. According to section 2(t), a person with disability means “a person suffering from not less than forty percent of any disability as certified by a medical authority”. The disabilities that have been listed in section 2 include blindness, low vision, hearing impairment, locomotor disability or cerebral palsy, mental retardation, mental illness and persons cured of leprosy. In addition, autism and multiple disabilities have been covered under the “National Trust for Welfare of Persons with Autism, Cerebral Palsy, Mental Retardation and Multiple Disabilities Act, 1999”.

### Prevention of disability

The law in its present form only exemplifies a narrow understanding of the right to health with emphasis on prevention, cure, improvement or elimination of disability, which in any case are conditions that generally exist before the onset of disability and therefore have no logical connections to the rights of those who are disabled. For example, section 25 of the Persons with Disabilities Act, 1995 states as follows: “Within the limits of their economic capacity and development, the appropriate Governments and the local authorities with a view to preventing the occurrence of Disabilities shall (a) undertake or cause to be undertaken surveys investigations and research concerning the cause of occurrence of disabilities; (b) promote various methods of preventing disabilities; (c) screen all the children at least once in a year for the purpose of identifying ‘atrisk’ cases; (d) provide training facilities to the staff at the Primary Health centers; (e) sponsor or cause to be sponsored awareness campaigns and disseminate or cause to be disseminated information for general hygiene, health and sanitation; (f) take measure for prenatal, peri-natal and post-natal care of mother and child; (g) educate the public through the pre-school primary health centers, village level workers and anganwadi workers; and (h) create awareness amongst the masses through television, radio and other mass media on the causes of disabilities and the preventive measures to be adopted”.[6]

As certain groups among the disabled are more vulnerable than others, a special enactment for the protection of such persons, their property and well-being was felt necessary. The enactment of the National Trust for Welfare of Persons with Autism, Cerebral Palsy, Mental Retardation and Multiple Disabilities Act 1999 (referred to as the National Trust Act) aims to fulfill a common demand of families seeking reliable arrangement for their severely disabled wards. The specific objectives of the act are:

1. to enable and empower persons with disabilities to live as independently and as fully as possible within and as close to the community to which they belong,
2. to promote measures for the care and protection of persons with disability in the event of death of their parent or guardian and
3. to extend support to registered organisations to provide need-based services during the period of crisis in the family of disabled covered under this act.

The National Trust Act mandates the creation of a local level committee (LLC) comprising District Magistrate along with one representative from a registered organisation and one person with a disability

### The Rehabilitation Council of India Act, 1992

The Rehabilitation Council of India (RCI) was set up by the Government of India in 1986 initially as a society to regulate and standardise training policies and programmers in the field of rehabilitation of persons with disabilities. The urgent need for minimum standards was felt as the majority of persons engaged in education, vocational training and counselling of persons with disabilities were not professionally qualified. Poor academic and training standards adversely affect the chances of disabled succeeding in the world of work. Therefore, an act of parliament in 1992 enhanced the status of the council to a statutory body with the following aims:

1. To standardise training courses for professionals dealing with people with disabilities,
2. To prescribe minimum standards of education and training for various categories of professionals dealing with people having disabilities,
3. To regulate these standards in all training institutions uniformly throughout the country,
4. To promote research in rehabilitation and special education and
5. To maintain Central Rehabilitation Register for registration of professionals.

The RCI regulates training standards for 16 categories of rehabilitation workers. The Council is pro-actively promoting training and research initiatives utilising experience of specialised as well as mainstream academic institutions.

Ministry of Social Justice and Empowerment had amended section 47 of the Persons With Disability (PWD) Act regarding non invalidation of the government servant who has been permanently incapacitated on account of mental or physical disability vide order 6/17/2002-SCS/1925 and states that no establishment shall dispense with or reduce in rank an employee who acquires a disability during his service, and the employee who has acquired disability, if not found suitable for the post he was holding, could be shifted to some other post with the same pay scale and service benefits. In case it is not possible to adjust him against any post, he may be kept on supernumerary post until a suitable post is available or he attains the age of superannuation, whichever is earlier. No promotion shall be denied to a person merely on the ground of his disability.

According to the persons with Disabilities Act rules, Ministry of Social Justice and Empowerment issued a gazette notification on June 1, 2001.[7] Here guidelines for evaluation of the following disabilities and process of certification are outlined: (i) visual disability, (ii) locomotor/orthopaedic disability, (iii) speech and hearing disability, (iv) mental retardation and (v) multiple disabilities. Medical Board duly constituted by the central and state governments can only issue disability certificate. The state government can constitute a medical board consisting of at least three members out of whom at least one shall be a specialist in the particular field, as the case may be. The Director General of Health Services, Ministry of Health and Family Welfare will be the final authority, should there arise any controversy/ doubt regarding the interpretation of the definitions/ classifications/evaluation tests, and the like.

## CONCLUSIONS

All major burn cases have to be notified to police. There are procedural differences about notifying as well as issuing certificate of death. Quantifying the Permanent Physical Impairment and issuing authenticated certificate for the same has been facing cross questions from the advocates. There is limited understanding of the differences between physical impairment and disability, amongst medical and non-medical fraternity. Medical personnel are empowered by the MCI act to issue certificate of Permanent Physical Impairment based on the guidelines prevailing in the country at present. It then becomes the duty of the law or compensation authorities to calculate loss of earning capacity of the person. Guidelines for calculating physical impairment in burns of head and neck, face, trunk and genitalia are based on the area and depth of involvement and calculation can be so based easily in arriving at the percentage of permanent physical impairment of a particular anatomical component of the body. There are several efforts at both the national and international levels to help and support disabled persons, and burn survivors would qualify for several of these services.

## DISCLAIMER

Foregoing presentation is personal view of the author, based on his personal experience. Any future legal amendments may change these views.

## References

[1] (2000). American Medical Association’s Guides to the evaluation of permanent impairment-American Medical Association.

[2] http://indiacode.nic.in/fullact1.asp?tfnm=192308.

[3] http://indiacode.Nic.In/fullact1.Asp?Tfnm=200046.

[4] (1982). The Manual for Doctors to evaluate Permanent Physical Impairment- DGHS.

[5] (1996). Evaluation of Impairments, Disabilities in case of Occupational and other Diseases and Accidents-Society for Participatory Research in India.

[6] http://socialjustice.nic.in/disabled.

[7] (1995). Pwd act, 1995: The persons with disabilities (equal opportunities, protection of rights and full participation) act.

